# Who Has Active Lifestyles? Personality and Activity Diversity in Adulthood

**DOI:** 10.1093/geroni/igab046.962

**Published:** 2021-12-17

**Authors:** Soomi Lee, Susan Charles, Karen Fingerman, David Almeida

**Affiliations:** 1 University of South Florida, Tampa, Florida, United States; 2 University of California, Irvine, Irvine, California, United States; 3 The University of Texas at Austin, Austin, Texas, United States; 4 Pennsylvania State University, University Park, Pennsylvania, United States

## Abstract

Broad and even participation across daily activities (“activity diversity”) has been found to be associated with better health. Less is known about who has greater activity diversity. We examined whether personality traits are associated with activity diversity in two independent samples of adults. Data came from the Midlife in the United States Study II and Refresher (n=2623, Mage=54yrs) and Daily Experiences and Well-being in Late Life Study (n=308, Mage=74yrs) who responded to daily activity questions. We constructed activity diversity scores in each sample using Shannon’s entropy. We focused on three personality traits -- conscientiousness, extraversion, neuroticism -- often associated with health. Higher extraversion was associated with greater activity diversity, replicated across the two samples. The associations were independent of conscientiousness and neuroticism (both were not significant), total activity time/frequency, age, gender, race, education, and self-rated health. Results suggest that future activity interventions may need to target those with lower extraversion.

